# Scaling up Ghana's national newborn care initiative: integrating 'helping babies breathe' (HBB), 'essential care for every baby' (ECEB), and newborn 'infection prevention' (IP) trainings

**DOI:** 10.1186/s12913-020-05225-2

**Published:** 2020-08-12

**Authors:** Margaret Amanua Chinbuah, Mira Taylor, Magdalena Serpa, Goldy Mazia, Patience Korkor Cofie, Williams Kwarah, Suzanne Dawson, Brett D. Nelson, Cyril Engmann

**Affiliations:** 1 PATH, Accra, Ghana; 2 Koforidua Regional Hospital, Eastern Region, Ghana Health Services, Koforidua, Ghana; 3 PATH, DC, Seattle, USA; 4 One Health Institute, Colorado State University, Colorado, CO USA; 5 Save the Children, Westport, USA; 6 Divisions of Global Health and Neonatology, Department of Pediatrics, Massachusetts General Hospital, Boston, MA USA; 7 Harvard Medical School, Boston, MA USA; 8 Departments of Pediatrics and Global Health, Schools of Medicine and Public Health, University of Washington, Seattle, USA

## Abstract

**Background:**

Responding to stagnating neonatal mortality rates in Ghana, a five-year collaboration called Making Every Baby Count Initiative (MEBCI) was undertaken to improve the quality of newborn care provided around the time of birth. A multi-pronged approach was used to build health worker (HW) capacity in resuscitation, essential newborn care, and infection prevention using a curriculum built on the American Academy of Pediatric’s (AAP) Helping Babies Breathe (HBB) and Essential Care for Every Baby (ECEB) modules with an added section on infection prevention (IP).

**Methods:**

MEBCI used a training of trainer’s approach to train 3688 health workers from district-level facilities in four regions in Ghana between June 2015 and July 2017. Prior to training, HWs familiarized themselves with the learning materials. Concurrently, MEBCI worked to improve enabling environments that would sustain the increased capacity of trained health workers. Knowledge and skills gained were tested using AAP’s Knowledge checklist and validated single-scenario Objective Structured Clinical Examinations (OSCEs) tools.

Findings: Majority of HWs trained were midwives (58.8%) and came from district-level hospitals (88.4%). Most HWs passed the HBB OSCE (99.9%, 3436/3440). Age of doctors was negatively associated with HBB scores (*r* = − 0.16, *p* = 0.0312). Similarly, older midwives had lower HBB scores (*r* = − 0.33, *p value* < 0.001). *Initiating ventilation within the Golden Minute* was challenging for HWs (78.5% passed) across all regions. Overall, the pass rate for ECEB OSCEs was 99.9% in all regions. *Classify newborn for further care* and *communicate plan to family* were frequent challenges observed in Volta Region (69.5% and 72.0% pass rate respectively). HWs less than 40 years of age performed significantly better than health workers older than 40 years *(p =* 0.023). Age of only paediatricians was positively associated with ECEB scores (*r* = 0.77, *p* < 0.001) while age of midwives was negatively associated with ECEB scores (*r* = − 0.08, *p* < 0.001).

**Conclusion:**

MEBCI’s integrated HBB-ECEB-IP training resulted in significant mastery of the clinical knowledge and skills of HWs. Harmonization and standardization of the course delivery by trainers and having a core team to ensure training fidelity are essential to maintaining high quality while scaling a program nationally.

**Funding:**

Children’s Investment Fund Foundation (CIFF).

## Background

Over the past three decades, progress in reducing newborn mortality worldwide has stagnated compared to reductions in maternal and under-five childhood mortality [1, 2]. Estimates of global neonatal mortality rates (NMR) indicate a fall of 49% over almost two decades, from 37 deaths per 1000 live births in 1990 to 19 deaths per 1000 live births in 2016 [2]. Based on current estimates, NMR constitutes 46% of the total under-five mortality worldwide [2]. The leading causes of neonatal deaths are complications associated with prematurity (35%), intrapartum-related deaths including birth asphyxia (24%), and infection (22%) [2, 3]. The relatively slow progress in reducing neonatal mortality over the years compared with under-five mortality led the World Health Organization, UNICEF, and other partners to develop and launch the Every Newborn Action Plan (ENAP) in 2014. The ENAP attempts to focus global efforts on improving perinatal health and survival [4, 5].

The plight of the newborn globally is mirrored in Ghana. NMR experienced near stagnation at 47.9 per 1000 live births from 1988, when population-level measurements began, through the mid-2000’s [6]. Over the past decade, there has been a modest decrease, with the 2014 Demographic Health Survey estimating NMR at 29 per 1000 live births [6]. In 2013, bottleneck and situational analyses of health system issues affecting newborn care in Ghana identified various barriers at national and regional levels [7]. Key among these were significant deficits in the knowledge and skills of health workers (HWs). Consequently, a Ghana National Newborn Health Strategy and Action Plan (GNNHSAP) for 2014–2018 was developed to guide Ghana’s efforts to reduce perinatal deaths [7]. The GNNHSAP aimed to reduce NMR from 32 per 1000 live births in 2011 to 21 per 1000 live births in 2018. A key component of GNNHSAP was to build HWs capacity to provide high-quality, essential newborn care. Ample evidence from the peer-reviewed literature indicates that improvements in these domains positively affect newborn outcomes [8–13].

PATH and Kybele, two non-governmental organizations, working together with the Ghana Health Service (GHS) and with funding support from the Children’s Investment Fund Foundation, implemented a five-year Making Every Baby Count Initiative (MEBCI) to improve the quality of newborn care provided around the time of birth. This paper describes the design and implementation of MEBCI’s innovative training approach to improve district-level HWs’ knowledge and skills in newborn resuscitation, essential newborn care, and newborn infection prevention.

## Methods

### Country context

Ghana is a low-middle-income country situated along the Gulf of Guinea in West Africa. It has a total population of nearly 28 million [14]. At the time of implementing this initiative, there were ten administrative regions in the country, with a relatively centralized national hub. Ghana operates a free healthcare system for mothers and their children under the age of five, although there remain inequities in healthcare provision due to geography, gender, and socio-economic background.

### The MEBCI program

The MEBCI program was started in Ghana in September 2013 with a focus on strengthening national leadership in newborn health and building capacity in newborn care at regional (led by Kybele-GHS) and district-level (led by PATH-GHS) facilities in four regions: Brong Ahafo (BAR), Eastern (ER), Volta (VR), and Ashanti (AR) regions. Together, these regions constitute about 58% of the nation’s population and were purposively selected because of their exceptionally high NMRs [15, 16].

Nationwide, close to 60% of deliveries occur at the regional and district-level hospitals [17]. Therefore, MEBCI strategically devised a plan with GHS to focus training in all regional hospitals and district-level hospitals across the target regions. Where districts had no hospitals, large health centres were selected. All HWs who conducted deliveries or provided other aspects of newborn care were enrolled on a rotating basis into one of the training cohorts.

The initiative developed a training approach targeting the three main causes of neonatal mortality: complications of prematurity, intrapartum-related events, and infections. To increase the likelihood of MEBCI’s success, PATH worked with GHS to create an enabling environment. Key elements of this environment included developing a list of essential newborn commodities (including practice mannequins and training documents for each facility); improving data collection and management (in addition to revising the delivery register and including key indicators in national database); training midwives, health information officers, public health nurses, and other data collectors to accurately document and collate data; conducting sensitivity training for facility management; and performing advocacy activities targeting community and religious leaders.

### The MEBCI training approach

#### Engagement of the national public health system

To ensure sustainability, the MEBCI program was fully embedded within the existing public healthcare delivery system at the national, regional, and district levels. In consultation with the Family Health Division and the Institutional Care Division of the GHS, PATH developed a training approach consisting of six steps: 1) national-level planning, 2) selection and orientation of national experts, 3) selection and training of Master Trainers, 4) selection and training of Regional Trainers, 5) implementation of HW trainings, and 6) implementation of supportive supervisory follow-up visits of the trained HWs.

### Step 1. National-level planning

#### Training package development

To build health care provider clinical capacity in essential newborn care, PATH, in close collaboration with GHS adopted two curricula from the American Academy of Pediatrics’ (AAP) Helping Babies Survive strategy, namely the Helping Babies Breathe (HBB) and Essential Care for Every Baby (ECEB) training programs both of which have improved NMR. In addition, a module for Infection Prevention (IP) including reprocessing resuscitation equipment, was developed using material adapted from various sources [18–21]. As recommended by the AAP, a stakeholder consensus meeting was held with local experts for localization and adaptation of the course material to conform to GHS guidelines and recommendations [22, 23].

#### Estimating and procuring the newborn equipment

We estimated and procured the following educational material and equipment for basic neonatal resuscitation: Laerdal’s HBB and ECEB educational materials, bag-mask devices, reusable bulb suction devices, and NeoNatalie™ newborn simulators for conducting the trainings, post-training clinical practice, and conducting deliveries in the participating facilities [24].

#### Front-line trainers trained

In each target region, all HWs working in the maternity unit (i.e., the labour ward, post-partum ward) as well as antenatal clinics, the operating theatres in facilities conducting caesarean sections, and wards where inpatient care is provided to sick newborns at district-level health facilities or the selected large health centres were listed and identified for training. These staff comprised of midwives, paediatricians, medical officers, anaesthetists, obstetricians, paediatric nurses, critical care nurses, medical/physician assistants, community health nurses and general nurses working with newborns. In a few facilities where staff shortage was acute, other cadres such as ward assistants and enrolled nurses who conducted deliveries were also included in the trainings. After the first training, hospital managers, executive-level nurses (Deputy Director of Nursing Services), and maternity unit head nurses were invited to participate in the training to enable them to support changes to improve facility’s readiness in providing newborn care services. Regional hospital staff trained by the Kybele-GHS teams are not included in this paper.

### Step 2. Selection and orientation of national experts

#### National level

In October 2014, six paediatricians and one senior midwife, local experts in maternal and newborn care, were identified by the GHS and asked to join the PATH team as national facilitators. These seven, together with two members of the GHS’s leadership from the Family Health and Institutional Care Divisions, led by two PATH staff with expertise in global newborn health programming met over 5 days to review and revise teaching materials and presentations to suit the local context.

### Step 3: selection and training of master trainers

The Family Health Division and Regional Health Administration of the GHS identified 30 HWs in the country who were experienced in child health trainings to be trained as Master Trainers. HWs (paediatricians, midwives, nursing tutors, obstetricians, and anaesthetists) from other regions, who had previously been involved as trainers in other child-related programs, were purposively identified by the GHS and were invited to join the program. To include all relevant cadres and health care levels, we also purposively selected and invited staff from our four regional hospitals, four pre-service training institutions (one in each of the target regions), and two of the public medical schools and district hospitals.

Following this, the seven National Facilitators led by the PATH newborn care experts conducted a Training of Trainers (TOTs) for these 30 prospective Master Trainers. Each nominated HW had received the AAP’s HBB learner workbook and ECEB provider guide 2 weeks before the training. Master Trainers were trained in HBB (two days), ECEB (two days), and IP (one day). The goal of this five-day centralized training was to be thorough, systematic and to ensure full knowledge and skill transfer. On the last day of the course, about two and half hours was dedicated to teaching and practicing training and facilitation skills by Master Trainers. After the training, each Master Trainer received a Laerdal’s NeoNatalie™ newborn mannequin, and facilitator sets for HBB and ECEB to aid personal practice and participation in subsequent regional trainings [24]. For IP, master trainers relied on training resources shared.

### Step 4: selection and training of regional trainers

Based on feedback received during the master training, a decision was made to further improve the presentations and mode of communication, to ensure clarity, and to communicate more effectively to the wide range of HWs. Over 3 days, the PATH-GHS team deliberated on how to improve the content and flow of presentations. Since there were no practical clinical sessions, the World Health Organization’s Integrated Management of Childhood Illness (IMCI) videos [25] on counting respiratory rate and recognizing chest in-drawing in a newborn as well as Global Health Media Project [26] videos on danger signs and breastfeeding were added to the relevant training sessions, aiding in the teaching of these concepts. To make IP sessions more engaging and to give the midwives leadership roles, some presentations were changed to demonstrations, led by a midwife in full personal protective equipment. The time for practice and role-play exercises was increased. These changes, however, extended the training by half a day.

To initiate regional activities, discussions were held with the Regional Health Administration and the heads of targeted health facilities to introduce them to the initiative, receive input, and address any concerns regarding project implementation in the regions. Because the state of newborn care varied widely within each region, we also conducted a rapid health assessment of all participating health facilities prior to program implementation in each region using a standardized assessment tool, adapted from material from various sources [18, 27–30]. Simultaneously, each Regional Health Administration were asked to purposively select from their regions 20–30 HWs whose work involved newborn care, to be trained as Regional Trainers. We requested a mix of paediatricians (where available), midwives, medical officers, paediatric nurses, and anaesthetists from the GHS and one tutor each from a nursing/midwifery training institution in the target regions.

### Step 5: implementation of HW trainings

#### Planning at the regional level

Members of the Regional Health Administration were taught how to plan for and organize the trainings. In BAR, where training was first initiated, some of the nominated Regional Trainers could not initially deliver their sessions effectively during practice and were excluded from further trainings sessions. Learning from BAR, the core PATH/GHS training team introduced criteria for selection of Regional Trainers (e.g., strong clinical skills, articulate, and passionate about newborn care), and the concept of “potential” Regional Trainers in the subsequent three regions. Selection for participation in the TOTs was no longer an automatic indication for designation as a MEBCI Regional Trainer, but only if a certain mastery level was gained.

#### Preparatory meeting and regional TOT

We preceded each training with a three-day preparatory meeting to hone the training facilitation skills of the Regional Trainers. The objective of the preparatory meeting was to assign roles and responsibilities and to discuss presentation styles, to include the acquisition and practice of facilitation skills required for presentation, demonstration, role play, leading group discussions, conducting the Objective Clinical Structured Examinations (OSCEs), standardization of scoring, and review of all training materials. The potential Regional Trainers received one-on-one coaching for 1–2 hours, learning how to demonstrate concepts using the AAP educational materials and mannequins in a systematic manner. Potential Regional Trainers then presented their sessions before the group and received immediate feedback. As the competency of Regional Trainers in delivery of the training improved, the duration of the preparatory meeting was reduced from 3 days for the first few trainings in each region to 1 day during subsequent trainings.

Once Regional Trainers had demonstrated sufficient ability to deliver the trainings and the Regional Health Administration had demonstrated sufficient ability to plan and organize the trainings, the core PATH/GHS training team moved on to the next region to initiate implementation activities. At that point, the Regional Trainers, supported by their Regional Health Administration, conducted the subsequent trainings in their regions unaccompanied.

##### Implementation of HW trainings

A list of all HWs (i.e., midwives, obstetricians, anaesthetists, paediatricians, medical officers, general nurses, paediatric nurses working in maternity wards, obstetric theatres and sick newborn units) was obtained from each of the participating facilities. HWs who had been purposively selected were invited to participate in the MEBCI trainings. Participation per training was limited to 6–8 facilities located near each other to facilitate supervision activities. Additionally, we also limited the number of HWs coming from any facility per training session to 6–15 staff depending on facility size and HW strength, in order to not adversely affect service provision. Thus, each facility sent multiple cohorts of HWs for training until all their identified HWs was trained. By doing so, multiple cohorts of 36–48 HWs from various units in 6–8 facilities were trained per session until all those identified had been trained. Each trainee received the reading materials 2 weeks ahead of trainings with an instruction to read the material and complete the self-check exercises.

**Fig. 1:**
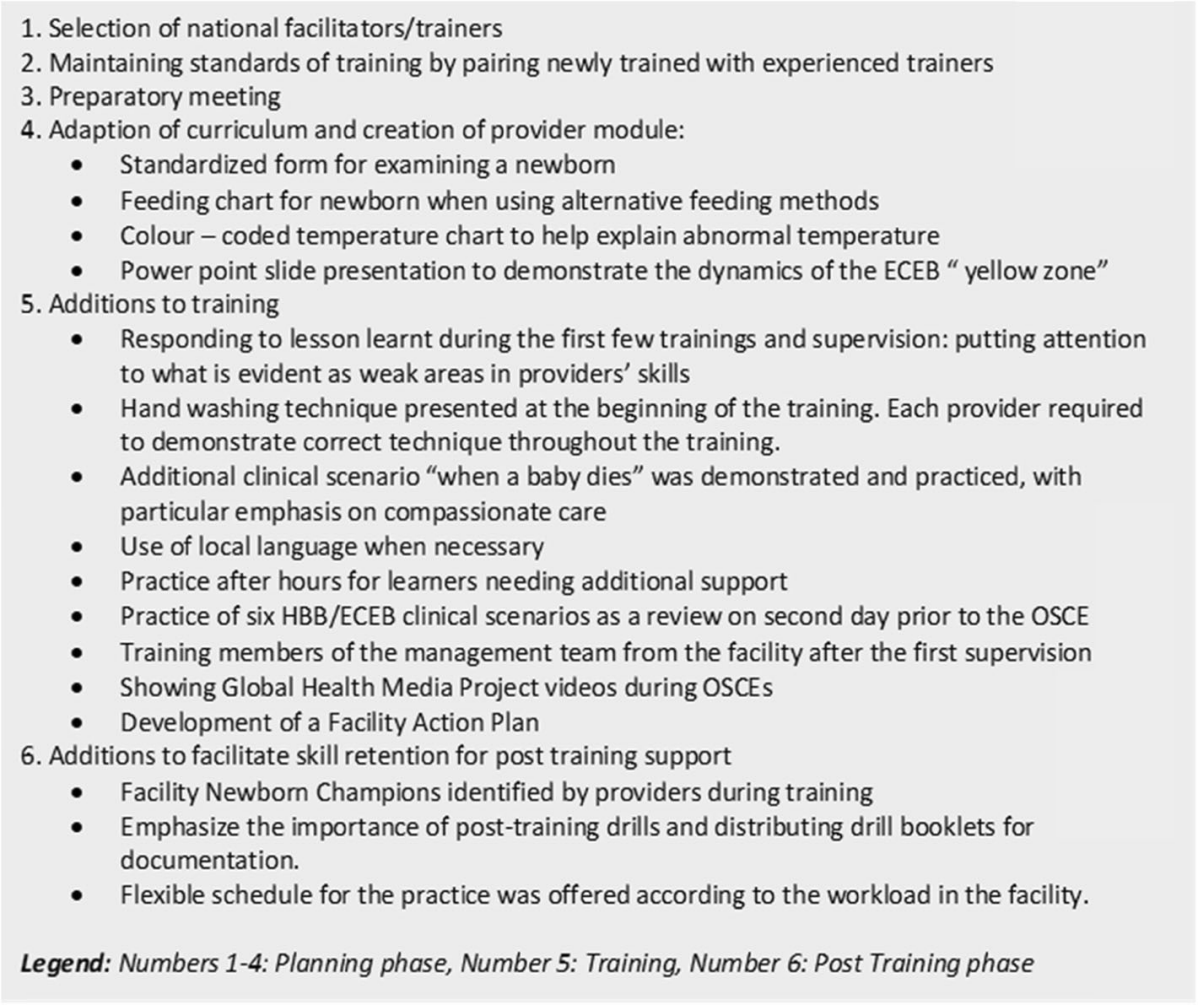
Key features of the MEBCI training

We kept fidelity with AAP training guidelines, including having a maximum ratio of six providers per facilitator. Facilitators closely followed HBB and ECEB facilitator flip charts. Each exercise was demonstrated by the facilitators, followed by guided practice in HW pairs at the table. All six standardized case scenarios in the learning materials were demonstrated and practiced on Day 2 of HBB and ECEB. For the first few trainings in each region, National Facilitators and Master Trainers paired with Regional Trainers, thus continuing the one-on-one coaching initiated during the preparatory meetings. Often, seasoned facilitators presented and demonstrated concepts in the first training, while newer trainers observed and improved their skills before taking on ensuing trainings. We developed other aids to improve uptake of the material. For example, since ECEB had not been launched in Ghana at the time, we had to create our own feeding charts. Group discussions, held after each practice session, were the basis for the development of facility-specific action plans at the end of the course for addressing gaps in service provision. Facilitator meetings were held at the end of each training day to discuss the provider performance, identify providers struggling to acquire new skills, assist those struggling, and give feedback to Regional Trainers on their performance. Key features of the training are shown in Fig. 1. In addition, and to ensure a facilitating environment, each facility was provided with basic resuscitation equipment for the labour ward, obstetric theatre, and the sick newborn wards/areas based on the estimates derived from a quantification tool developed by PATH [30]. After each training session, each HW in a training cohort received three post-training supervisory visits conducted by their training team over a period of 1 year.

### Knowledge and skills evaluation

#### HBB, ECEB and IP knowledge evaluation

Provider knowledge was ascertained before and after each training using AAP’s multiple-choice knowledge check for HBB [31] and ECEB [32] as well as a multiple-choice knowledge check for IP. The knowledge check for IP was developed by PATH and consisted of eight questions focusing on principles and components of newborn infection prevention in the institutional setting (e.g., standard precautions and prevention of hospital-acquired infection, risk factors, hand hygiene, and isolation).

#### HBB skills evaluation

The skills evaluation was completed using Laerdal’s NeoNatalie™ newborn mannequin. Every provider was required to successfully pass the bag-mask ventilation skill check scoring 7/7 before they were assessed using a validated 13-step HBB single-scenario OSCE [33] developed by the Tanzanian Ministry of Health and Social Welfare, Harvard Medical School, Children’s Investment Fund Foundation, and Jhpiego. The validated single-scenario HBB OSCE tool was used for its efficiency in large-scale skills evaluation of birth attendants who have been trained in neonatal resuscitation by the HBB program in low-resource countries. Trainees were expected to correctly perform the key steps on the mannequin to achieve a minimum score of 16 out of 23 to successfully pass the HBB OSCE.

#### ECEB skills evaluation

We administered a previously validated, 21-step, single-scenario ECEB OSCE jointly developed by PATH and Harvard Medical School but further adjusted by PATH and the GHS [34]. This validated single-scenario ECEB OSCE tool was designed to test the healthcare provider’s ability to deliver essential neonatal care and respond appropriately to evolving changes in the neonate’s condition using the ECEB colour-coded Action Plan. The green zone of the ECEB Action Plan guides the provision of routine care for a well newborn, the yellow zone guides the management of a newborn with a problem, and the red zone guides the management of a newborn needing advanced care. The provider had to achieve a minimum score of 20 out of 28 to successfully pass the ECEB OSCE.

#### IP skills evaluation

Evaluation of the IP skills was integrated within the HBB OSCE (e.g., donning of gloves, use of sterile cot sheets, delivery of the newborn with double gloves with outer gloves to be removed before cutting the umbilical cord, use of exclusive cord cutting scissors) and the ECEB OSCE (e.g., hand washing before and after touching the newborn and before and after each procedure, safe injection practices, appropriate sharps disposal, cleaning of the weighing scale and thermometer before and after each use).

### Knowledge and skills acquisition goals

Our primary goal was the acquisition of knowledge and skills for all three courses (HBB, ECEB, and IP). Acquisition of knowledge was defined as all providers achieving a passing score of 80% on each of the post-training knowledge tests for HBB, ECEB, and IP.

#### HBB skills acquisition goal

Our primary goal for HBB skills acquisition was for all providers to pass the HBB OSCE (a score of 16/23 or more) and for 80% of providers to initiate effective ventilation within the *Golden Minute*™, the critical first minute after birth during which neonates should begin breathing spontaneously or receive assistance with adequate and effective bag-mask ventilation [35]. Our secondary goal of interest was that 80% of providers would be able to 1) dry the newborn thoroughly, 2) clear the airway and stimulate breathing, 3) ventilate at a rate of 40–50 breaths per minute, and 4) demonstrate in correct sequence the steps to improve ventilation.

#### ECEB skills acquisition goal

Our primary goal for ECEB skills acquisition was for all providers would pass the ECEB OSCE defined as a score of 20/28 or higher. Our secondary goal of interest was that 80% of providers would: 1) correctly perform hand washing, 2) take steps to adequately prevent disease at birth (i.e., by correctly providing eye care, umbilical cord care, and vitamin K_1_), 3) correctly classify a newborn, and 4) correctly and promptly recognize a danger sign and act accordingly.

#### IP skills acquisition goals

No separate goals were set for IP skill acquisition instead IP was assessed as an integral part of specific steps for both HBB and ECEB.

### Data management and statistical methods

#### Data management

The results of the completed OSCE forms were entered into Microsoft Excel (Redmond, WA, USA) data sheets by trained regional monitoring officers in each of the four regions. The data were transmitted regularly to the PATH office in Accra for entry into a central database and cleaned using standard data cleaning guidelines. Periodic cross-checking of data in the database against the paper forms was done to ensure data quality and completeness.

#### Data analysis

Stata Statistical Software Version 12 (StataCorp LP, College Station, TX) was used for analysis. Data were analysed using descriptive and summary statistics. Background characteristics of the participants analysed included region, facility level, facility ownership, clinical cadre, gender, age, and clinical experience. The means and standard deviations (SD) for age and clinical experience of participants were calculated. Age and clinical experience were further categorized into groups. Chi-squared test of proportions were used to test differences in pass rates while Pearson correlations coefficient was used to test associations between age, length of practice and OSCE scores at 95% significance level.

## Results

**Fig. 2:**
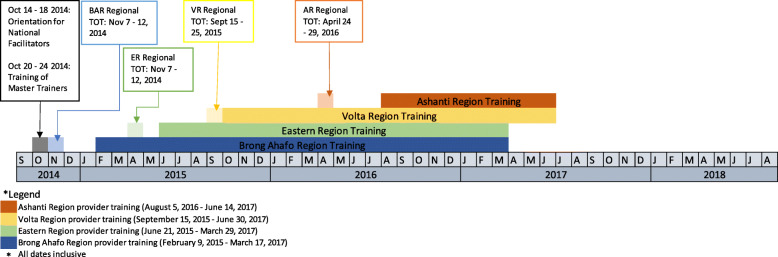
Timeline for sequential roll-out of MEBCI capacity building activities

Capacity-building activities started at the national level in October 2014 and was followed by regional-level activities in the four target regions. A timeline of the major activities is presented in Fig. 2.

Between February 2015 and July 2017, a total of 3688 HWs from 99 districts-level hospitals, 48 health centres, and 4 polyclinics were trained. In some regions, a few HWs from non-targeted facilities were trained at the request of the Regional Health Administration but these were not followed over time. In addition, some members of hospital management teams (e.g., medical directors, deputy directors of nursing services, ward-charges) were also trained to understand and support the program implementation and facilitate change in their respective health facilities.

### Demographics

The median age of the 3688 HWs trained was 30.0 years (SD 14.9, range 20–69 years). Table 1 shows the distribution of HWs across different demographic variables. Most trainees were midwives (58.8%, 2170/3688) and came from district-level hospitals (88.4%, 3261/3688). The average length of trainees’ clinical experience was 6.5 years, (SD = 8.0, range < 1 - 43 years). Members of hospital management teams, who constituted less than 2% of participants, were excluded from further analysis. The primary focus of this article is on the process of implementation of the integrated approach and the overall performance of the 3440 trained HWs as well as performance within the four target regions.

**Table 1:** Demographic characteristics of all training participants by region

	BAR ***n*** = 839	ER ***n*** = 853	VR ***n*** = 560	AR ***n*** = 1463	^**a**^Total ***N*** = 3688
**Age group:**					
Less than 30 years	435 (51.9)	376 (44.1)	282 (50.4)	652 (45.4)	1745 (47.3)
30–39 years	256 (30.5)	289 (33.9)	183 (32.7)	522 (36.4)	1250 (33.9)
More 40 years	122 (14.5)	126 (21.5)	91 (16.2)	167 (11.7)	563 (15.3)
**Gender:**					
Male	149 (17.8)	106 (12.4)	124 (22.1)	205 (14.3)	584 (15.8)
Female	690 (81.2)	747 (87.6)	436 (77.9)	1231 (85.7)	3104 (84.2)
**Cadre:**					
Paediatrician/ Obstetrician	6 (0.7)	9 (1.0)	3 (0.6)	14 (1.0)	32 (0.9)
Anaesthetist	43 (5.1)	54 (6.3)	35 (6.3)	62 (4.3)	194 (5.5)
Midwife	486 (57.9)	587 (57.1)	326 (58.2)	871 (60.7)	2170 (58.8)
Medical Officer	56 (6.7)	46 (5.4)	49 (8.8)	42 (2.9)	193 (5.2)
General Nurse	98 (11.7)	187 (21.9)	107 (19.1)	250 (17.4)	642 (17.4)
PN/CN/EmN/PON	8 (1.0)	10 (1.2)	2 (0.4)	20 (1.4)	40 (1.08)
EnN/HA/WA	97 (11.6)	8 (0.9)	16 (2.9)	117 (8.2)	238 (6.5)
CHN and PHN	13 (1.6)	28 (3.3)	9 (1.6)	16 (1.1)	66 (1.8)
MA/PA	16 (1.9)	19 (2.2)	19 (2.2)	18 (1.3)	5 (0.9)
RHD/DHD	16 (1.9)	5 (0.6)	8 (1.4)	22 (1.5)	51 (1.4)
**Clinical practice:**					
Less than 1 year	115 (13.7)	12 (1.4)	50 (8.9)	93 (6.5)	270 (7.3)
1–5 years	383 (45.7)	457 (53.6)	356 (63.6)	886 (61.7)	2082 (56.5)
6–10 years	130 (15.5)	174 (20.4)	68 (12.1)	236 (16.4)	608 (16.5)
More than 10 years	126 (15.0)	202 (23.7)	76 (13.6)	160 (11.5)	564 (15.3)
**Level of care:**					
District Hospital	759 (90.5)	732 (85.8)	514 (91.8)	1256 (87.5)	3261 (88.4)
Polyclinic/Health Centre	67 (8.0)	105 (12.2)	43 (7.6)	167 (11.5)	380 (10.3)
RHD/ MHD/DHD	8 (1.1)	4 (0.5)	1 (0.2)	1 (0.1)	14 (0.3)
Regional Hospital	4 (0.5)	12 (1.4)	1 (0.2)	11 (0.7)	28 (0.8)
Training School	1 (1.1)	0 (0.0)	1 (0.2)	3 (0.2)	5 (0.1)
**Facility ownership:**					
GHS	376 (44.8)	553 (64.8)	358 (63.9)	992 (69.1)	2279 (61.8)
FBO/ Quasi-government	463 (55.2)	300 (35.2)	202 (36.1)	444 (30.9)	1409 (38.2)

^**a**^Missing data were less than 10% and have been excluded. RHD-Regional Health Directorate, MHD-Municipal Health Directorate, DHD-District Health Directorate. Community Health Nurses /Public Health Nurses = CHN/PHN; Enrolled Nurses, Health Assistants, and Ward Assistants = EN/HA/WA; Medical Assistants and Physician Assistants = MA/PA; Paediatric Nurses, Critical Care Nurses, Emergency Nurses, and Peri-operative Nurses = PN/CN/EmN/PON; Community-based Health Planning and Services compounds = CHPS; Ghana Health Service = GHS; faith-based organization health facilities = FBO. Health facilities supported by the government but managed privately = quasi-government facilities; Brong Ahafo Region = BAR, Eastern Region = ER, Volta Region = VR, and Ashanti Region = AR. Municipal Health Directorate = MHD, District Health Directorate = DHD, Regional Health Directorate = RHD

### Knowledge acquisition during training

Pre-reading of course materials were done by HWs ahead of the training. The pass mark was 80% on HBB, ECEB and IP knowledge test. The minimum number of HWs passing HBB, ECEB and IP at pre-test was 90.9, 87.7 and 83.7% respectively across the regions. The most challenging questions related to HBB were *delivery when there was meconium-stained amniotic-fluid*; *timing of cord clamping and cutting; ventilation with bag and mask; actions to take within the Golden Minute;* and *when to stop ventilation.* For ECEB, the most challenging questions were *managing hypothermia*; *cup feeding a newborn; recognizing severe jaundice;* and *recognizing danger signs.* For IP, *infection prevention practice in institutional setting in general* and *specifically in newborn units* were questions most HWs had challenges answering correctly*.* Post-test scores were significantly higher with improved pass rates (*p* < 0.001). Overall, the proportion of HWs passing was 99.7%, 99.6.7 and 98.7% for HBB, ECEB and IP respectively. Table 2 shows the results of HW performance on the pre and post knowledge test by region.

**Table 2:** Proportion of HWs who passed pre- and post-knowledge tests by region

		BAR	ER	VR	AR	Total
HBB	Pretest	94.6 (747/790)	90.9 (729/802)	97.8 (478/489)	92.2 (1253/1359)	93.2 (3207/3440)
	Posttest	99.2 (715/721)	99.9 (801/802)	100.0 (480/489)	99.8 (1355/1358)	99.7 (3360/3370)
ECEB	Pretest	87.7 (693/790)	90.7 (727/802)	90.8 (444/489)	88.4 (1200/1358)	89.1 (3064/3439)
	Posttest	98.7 (745/755)	100.0 (802/802)	100.0 (489/489)	99.7 (1354/1354)	99.6 (3390/3390)
IP	Pretest	85.5 (672/786)	83.7 (671/802)	84.0 (408/486)	86.3 (1170/1356)	85.2 (2921/3430)
	Posttest	97.5 (700/718)	99.6 (798/801)	98.0 (477/487)	99.0 (1340/1354)	98.7 (3315/3360)

#### Bag and mask skill acquisition

On the first attempt after training, 90.8% (3404/3089) achieved the passing score of 7/7 (100%) for the bag and mask skills test. Except for VR where 80.0% (366/460) passed, performance was good in other 3 regions: BAR 94.2% (742/788), ER 91.8% (735/801), and AR 92.0% (1246/1355). By the second attempt, 98.0% of the participants who failed on the first attempt had passed. The rest passed after a third attempt. The most challenging actions were checking the equipment for functionality before use; ventilating at the recommended rate; and performing additional steps to improve ventilation.

#### HBB skill acquisition

Almost all HWs (99.9%, 3436/3440) passed the HBB OSCE (obtained at least a score of 16 out of 23) on the first attempt after training, with little regional difference. There were no significant differences in pass rates with reference to gender, facility ownership, and level of care. However, in relation to age, younger HWs (< 40 years old) passed at higher rates compared to older HWs (> 40 years old) (90.0% vs 97.8%, *p* < 0.001). Age of medical doctors was negatively significantly associated with HBB scores (*r* = − 0.16, *p* = 0.03), older medical doctors had lower HBB scores. Similarly, older midwives had significantly lower HBB OSCE scores (*r* = − 0.33, *p* < 0.001). Age of General Nurses was also negatively significantly correlated with HBB OSCE scores (*r* = − 0.27, *p* < 0.001). Similarly, as the age of anesthetists increased, HBB OSCE scores decreased significantly (*r* = − 0.25, *p* < 0.001).

**Fig. 3:**
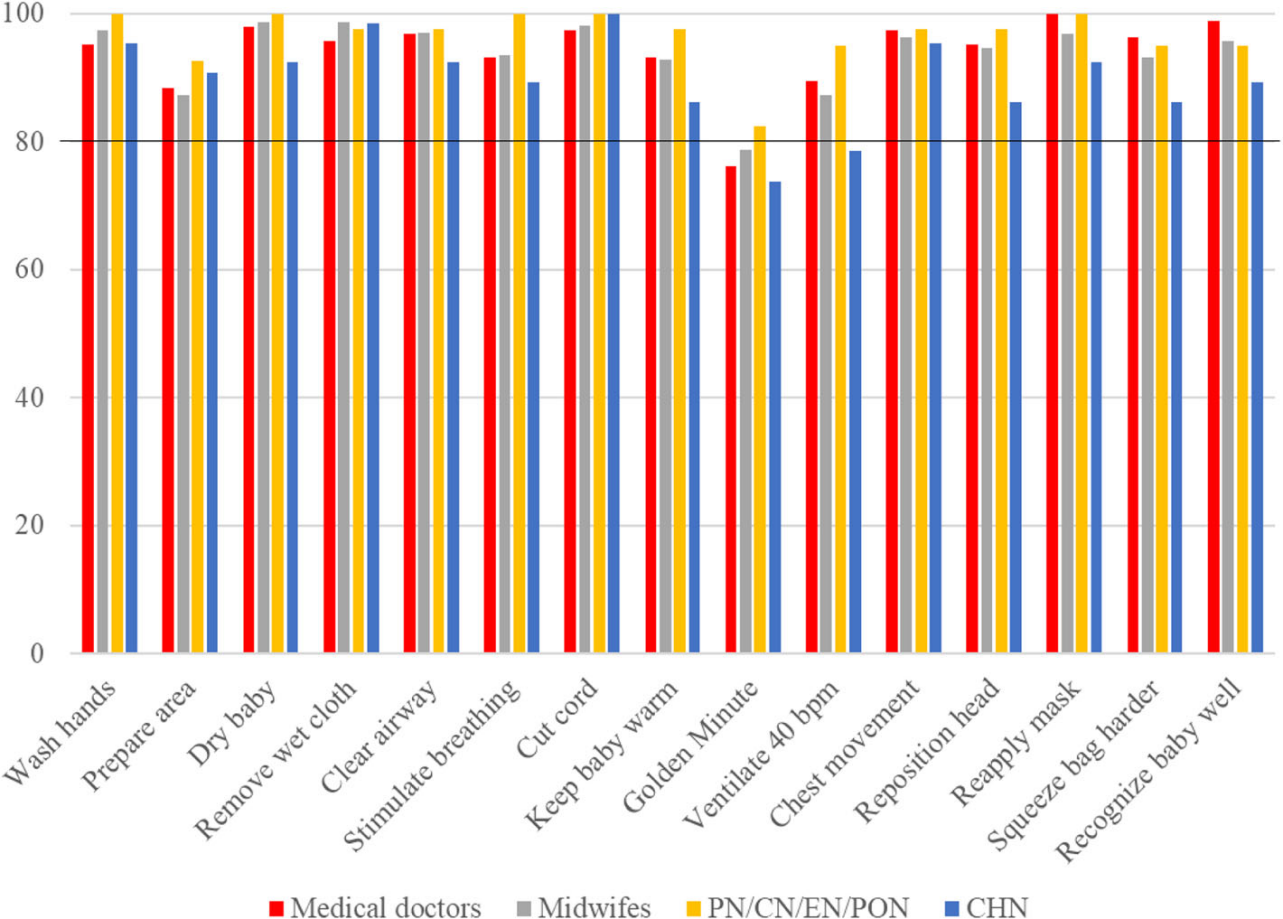
Performance of selected HW cadres on the HBB OSCE steps

**Fig. 4:**
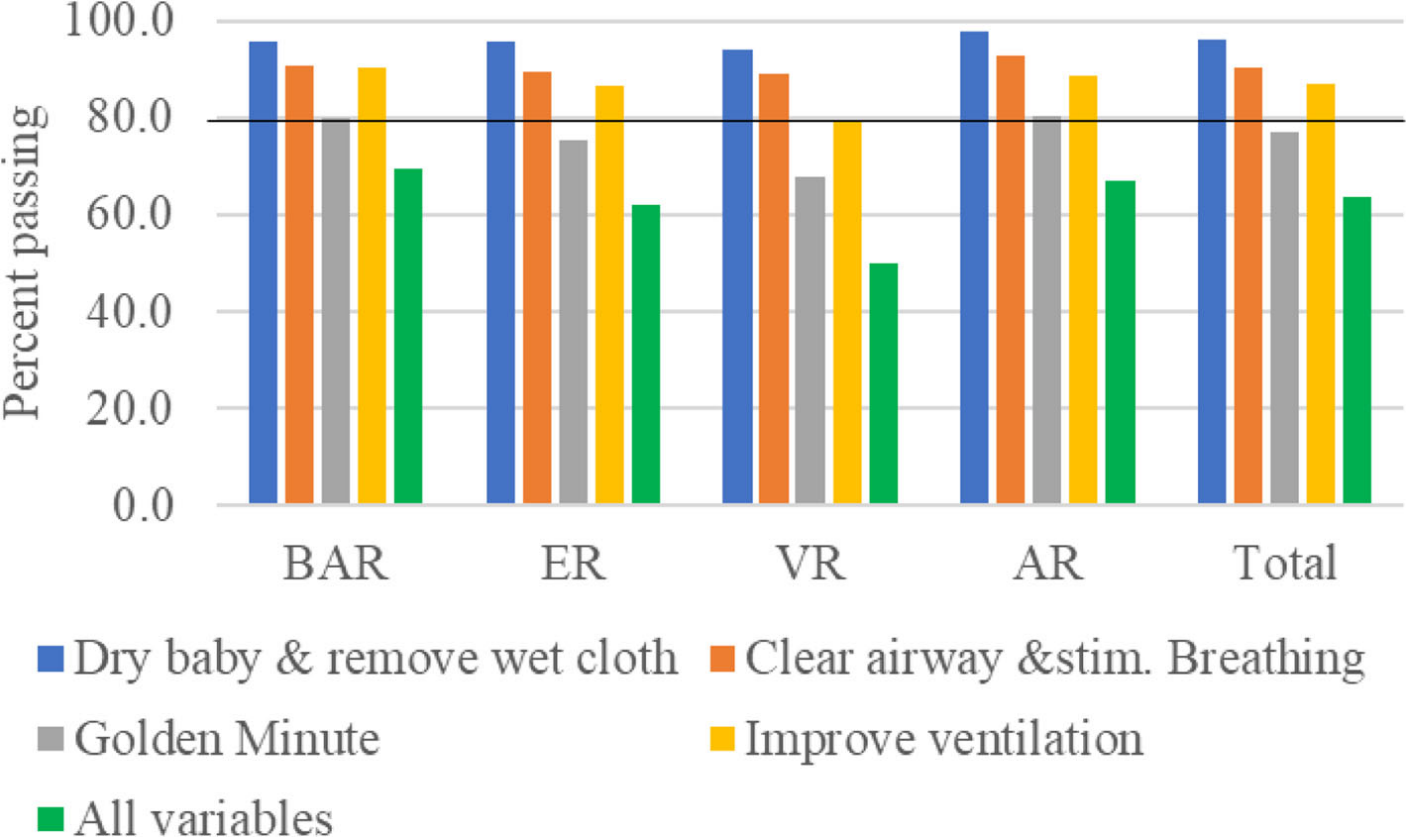
Performance of HWs on key steps in the HBB OSCEs

The performance of key clinical cadres on each of the HBB steps was compared and performance across regions was similar. Figure 3 shows the performance of medical doctors, midwives, critical care/emergency nurses, and community health nurses. Regional differences in HW performance on the secondary HBB goals are shown in Fig. 4. Again, performance by region was similar.

##### Clinical experience

The vast majority of HWs with less than 1 year of clinical experience passed the HBB OSCE on first attempt (98.9%). However, the pass rate declined with increasing clinical experience: 1–5 years (98.7%), 6–10 years (98.2%), 11–15 (97.2%), > 15 years (90.5%) (*p* < 0.001). Midwives with longer years of clinical experience had significantly lower HBB OSCE scores (*r* = − 0.28, *p* < 0.001). Similarly, anaesthetist with more years of clinical experience had a significantly lower HBB OSCE scores (*r* = − 0.17, *p* = 0.016).

Younger HWs and HWs with shorter clinical experience performed better than older HWs or HWs who had worked longer. For example, only 11.8% (319/2700) of HWs aged 40 years or more *initiated effective ventilation within the Golden Minute* compared with 88.2% (2381/2700) of HWs aged < 40 years (*p* < 0.001). Additionally, the ability of HWs to perform steps correctly declined with increasing length of service; 86.8% (231/266) of HWs with < 1 year of experience, 81.7% (1653/2024) of HWs with 1–5 years of experience, 78.8% (443/563) of HWs with 6–10 years of experience, 67.6% (98/145) of HWs with 11–15 years of experience, and 54.1% (70/314) of HWs with > 15 years of clinical experience performed the steps correctly.

Overall pass rates for critical care nurses, paediatric nurses and peri-operative nurses was 100%, midwives was 97.9%, general nurses managing newborns in special care units was 98.2%, community health nurses/public health nurses was 93.1%, and non-specialist medical officers was 99.3%. Correct performance for each step was also good. The most challenging step in all four regions was *initiating effective ventilation within the Golden Minute* (pass rate 78.5%), and in VR, *preparation for birth* and *correctly ventilate at a rate of 40–50 breaths per minute* (pass rate 69.9 and 77.5%, respectively).

### ECEB skill acquisition

**Fig. 5:**
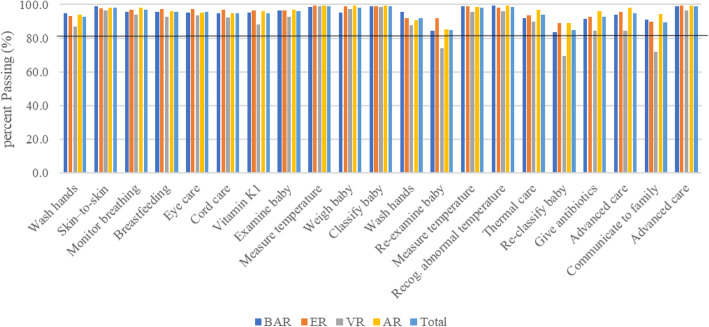
HW performance on the ECEB OSCE steps by region

The pass mark for ECEB was 20 out of 28 points. Overall, 99.9% (3438/3440) passed the ECEB OSCE. The exception was VR, where HWs had difficulty with specific steps: *classify the newborn for further care* (69.5%, 340/489) and *communicating the referral plan to the family* (72.0%, 352/489). Performance on the 21-step ECEB OSCE by region is shown in Fig. 5.

There was no significant difference in HW pass rate by gender, facility ownership, or level of care. In general, HWs less than 40 years of age performed better than HWs greater than 40 years of age (*p* = 0.023). Older midwives had significantly lower ECEB OSCE scores (*r* = − 0.08, *p* = 0.001), same as older Anaesthetist had significantly lower ECEB score (*r* = − 0.25, *p* = 0.006). However, we observed that age was positively and significantly associated with ECEB OSCE scores for paediatricians (*r* = 0.77, *p* < 0.001). Older paediatricians had higher ECEB OSCE scores.

Similarly, years of clinical experience of midwives was negatively associated with ECEB OSCE scores. Older midwives had lower scores (*r* = − 0.09, *p* < 0.001)., Older EN/HA/health assistants/ward assistants also had lower ECEB OSCE scores (*r* = − 0.25, *p* < 0.001). However, paediatricians with higher years of clinical experience had higher ECEB OSCE scores (*r* = 0.88, *p* < 0.001). Years of clinical experience of the other cadres of HW (medical doctors, general nurses, MA/PA, PN/CN/EN/PON, specialist and CHN/PHN) was not significantly associated with ECEB OSCE scores.

**Fig. 6:**
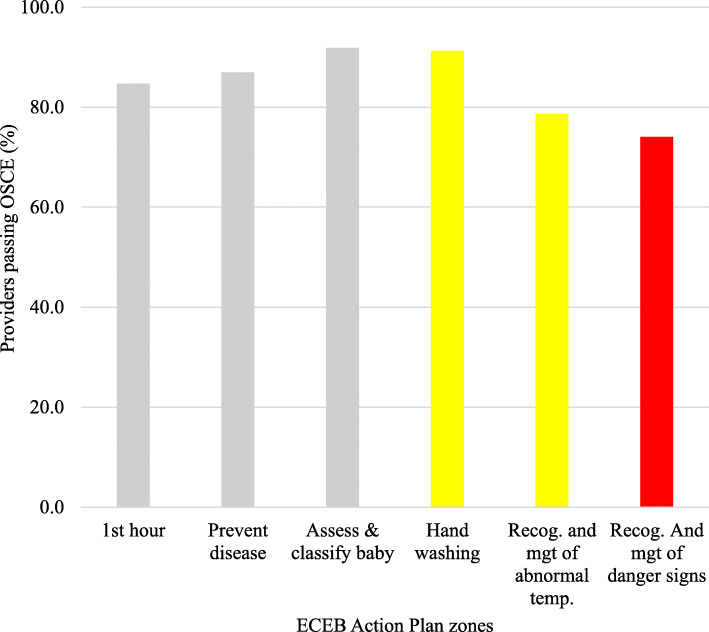
HW performance in relation to ECEB Action Plan zones

Steps that proved challenging for HWs 40 years and above were: *wash hands before re-examination* (4.9%), *re-examination of the newborn* (4.9%), and *classify the newborn for further care* (4.7%). The different HW cadres performed similarly on most ECEB OSCE steps. Community Health Nurses/Public Health Nurses had difficulty with *classifying the newborn for further care* (79.3%, 46/58) and *communicating the referral plan to the family* (75.9%, 44/58)*.* Paediatric Nurses/Critical care Nurses/Emergency Nurses/Peri-operative Nurses had difficulty *classifying the newborn for further care* (77.1%, 27/35). Figure 6 shows the HW pass rates for the ECEB OSCE steps related to the ECEB Action Plan colour-coded zones as well as for the secondary ECEB goals.

## Discussion

The Making Every Baby Count initiative in Ghana integrated the implementation of HBB, ECEB, and IP as a country-level package, aimed at addressing aspects of the three major causes of neonatal mortality at the primary and secondary healthcare level. MEBCI was not an implementation research initiative but a program targeted to scale-up interventions known to improve neonatal survival. The AAP Helping Babies Survive strategy has been shown by many others to improve HW knowledge and skills [36–39], but many of these studies have been limited to either HBB or ECEB as standalone studies. Our program also included Infection Prevention, which is crucial for improving neonatal survival. However, we excluded Essential Care for Small Babies (ECSB) in our package.

From the results, we achieved high knowledge acquisition. Test results (pre-post) were 93.2–99.7% for HBB, (89.0–99.6%) for ECEB, and 85.2–98.7% for IP. One potential explanation for this success was our insistence, in line with the AAP’s recommendation, that each HW receive the learning materials with the instruction to read and complete the “check yourself” exercises in their booklets 2 weeks before the training. Singhal et al. identified lack of learner workbook availability for study prior to the course as contributing to low HBB scores [40]. Pre-training availability of the learning materials may explain the relatively high pre-training scores found among HWs in our program. Nonetheless, HW knowledge improved significantly post-training for all the three courses.

Our primary goal was for all providers to pass the HBB OSCE (obtain a score of 16/23 or more) and for 80% of providers to initiate effective ventilation within the Golden Minute. Skill acquisition among the HWs was also high for HBB. After satisfactory performance on the bag-mask ventilation skills test (first attempt 90.8%, second attempt 98.0%), 99.9% of all trained HWs passed the single-scenario HBB OSCE. This is higher than reported in other studies [12, 35, 41, 42]. By increasing the time for practice during training sessions, HWs had sufficient time to learn the skills. Another contribution to the high HBB scores may have been the practice of all six clinical scenarios at the end of the course; we randomly called participants to demonstrate their response to different scenario’s, until there was a sense that most of the trainees had understood the algorithm sufficiently. During the course, a high standard for delivery of the training sessions was maintained through rigorous trainer preparation and maintaining fidelity to the training curricula. This may explain the minimal regional variation in our data.

Early initiation of basic resuscitation interventions including bag-mask ventilation may reduce intrapartum-related mortality in low-income countries [43]. Specifically, the risk for death and morbidity increased 16% for every 30-s delay in initiation of bag-mask ventilation up to 6 min (*p* = 0.045) in a study in Tanzania [43]. In the same study, more than two-thirds of the deaths occurred when ventilation was administered beyond 4 min [43]. Performance of the HWs in achieving the Golden Minute was suboptimal in our initiative, with only 78.5% of providers initiating effective ventilation within the Golden Minute™. Other studies have also reported that HWs had difficulty achieving the Golden Minute [12, 35, 38]. For many HWs, this was a new skill that needed to be learnt and correctly performed within a specific time. In our program, age appeared to influence performance on the Golden Minute. Fewer older HWs (67.6% of HWs aged 40–49 years and 47.9% of HWs > 49 years) achieved the Golden Minute compared to younger HWs (> 88% of HWs < 40 years). Achieving the Golden Minute was challenging for all cadres of HWs. However, critical care nurses performed better than midwives, physicians, and Community Health Nurses.

It is important that after training, clinical practice of various scenarios or drills is encouraged at the facility level to help improve or maintain resuscitation skills and timely decision-making [39]. HW pass rate for the HBB OSCE was also related to years of clinical experience. HWs with a shorter duration of clinical experience passed the HBB OSCEs at higher rates compared to older HWs and HWs with increasing clinical experience. This was also obvious during the training; many of the older HWs struggled with certain concepts and often had to receive extra attention. We speculate that this is may have been because older providers may have been more complacent, or that younger providers may have recently graduated from school, where they did more reading and were exposed to more recent evidence-based practices under direct supervision similar to our conduct of the OSCEs. This finding strengthens the need for continued education for HWs to maintain their knowledge and skills over time. The differences in knowledge and skill acquisition among different cadre we observed agrees with studies by Bang et al. [38].

Literature is scarce in describing the performance of HWs on various steps of the HBB OSCE. In their study, Arlington et al. described their most challenging steps as *preparing the area for ventilation*, *checking equipment for delivery*, and *stimulating breathing by rubbing the newborn’s back* [42]. In our initiative, the most challenging steps for HWs during training were preparation for birth (*preparing the area for ventilation and checking equipment for delivery)*, similar to their study and *achieving effective ventilation within sixty seconds of birth.*

ECEB is a more recent global training module. In a study evaluating uptake of ECEB, up to 10% of learners failed to achieve a passing score on one of the OSCEs [22]. Our primary goal for ECEB skill acquisition was that all providers pass the ECEB OSCE by obtaining a score of 20/28 or more [34]. HWs achieved a high pass rate (98.5%) for ECEB, likely because HWs were more familiar with many of the steps in ECEB. Our secondary goals of interest which was that 80% of HWs would correctly perform the following steps: 1) *hand washing* (92.9%), 2) adequate preventive disease (e.g., *providing eye care* (95.5%), *cord care* (94.9%), and *vitamin K*_*1*_ (94.8%)), 3) *initial classification of a newborn* (99.0%), and 4) prompt recognition of a *danger sign* (85.0%) was also achieved. However, a good proportion (15%, 517/3438) of HWs had challenges passing the *re-examine and reclassify the newborn for further care* step.

### Strengths, challenges, and strategies to address them

#### Strengths

We provided a comprehensive training package that addressed essential newborn care targeting the provision of neonatal resuscitation for intrapartum-related complications (birth asphyxia), some elements of care for the premature neonate, and basic infection prevention for prevention of neonatal infections– thus targeting the three major causes of neonatal mortality worldwide and in Ghana. Knowledge and skills of HWs were improved and maintained throughout the implementation period. The scores achieved across the four regions despite the large numbers of HWs trained and regional implementation could be a result of several aspects of our program design and implementation. We instituted a very systematic approach to training. The same core team (National Facilitators, the PATH newborn team, and a few Master Trainers) led the capacity-building efforts at the start of program implementation in each region.

We harmonized and maintained the quality and approaches of our trainers by ensuring a balance of strong facilitators in each training, assigning roles and responsibilities based on the abilities of trainers, allowing time for practice and correction during preparatory meetings, and standardizing the delivery across the initiative. Early in the training roll-out, we improved and modified the delivery of some specific algorithms. For example, we added a feeding chart for newborns, random quizzes for the different clinical scenarios for HBB and ECEB, and an animated PowerPoint presentation for explaining care of a newborn with abnormal temperature.

Training without systems strengthening is unlikely to make an impact. To ensure sustainability, working with the involvement of the GHS at the national, regional, and district levels right from the beginning was crucial for strengthening the health system and helping to ensure sustainability. Training the facility management (medical directors, matrons, and regional directors for clinical care) improved support for HWs to implement the newly acquired skills as well as improve the availability of the necessary equipment and other resources such as training mannequins. We involved a wide range of cadre as facilitators (midwives, nurses, anaesthetists, medical officers, obstetricians, and paediatricians) to facilitate adoption of essential newborn care and included some pre-service tutors from training schools in the target regions as well as two of our medical schools in an attempt to diffuse these skills into pre-service institutions. Although we recognize that this is unlikely to be enough on its own. The curriculum was adopted by the GHS to be rolled out to other regions nationally and into pre-service institutions.

#### Weaknesses and limitations of the program

The MEBCI trainings were centralized and costly (to be described in a future publication). Our experience with other trainings in the country shows that non-centralized training for such large numbers of HWs may have been difficult. However, in an ideal situation, a neonatal resuscitation training program for large-scale implementation in resource-limited settings may permit one-day courses if they are followed by frequent refresher or formal in-facility mentorship [44, 45]. The ideal duration of training remains unclear because studies show that frequent, short trainings are more effective than long, infrequent trainings. However, these are studies with smaller numbers of HWs. It is unclear if the same results would be achieved in a larger-scale, non-research setting such as ours [38, 46]. It would have been incredibly challenging logistically to train 3440 HWs and provide three follow-up visits within the timeframe allocated for the program without adopting a residential training approach. Centralized trainings also required frequently taking trainers away from their clinical work. After a while, some facility managers protested, leaving only about half of the Regional Trainers regularly available for further trainings.

There are limitations to our program. We did not evaluate long-term retention of knowledge in this program. As has been observed with other training programs, without additional intervention, some decrement in knowledge is expected to occur over time [38, 39, 42]. However, each trained HW was encouraged to use the AAP’s learner manuals and practice with the simulators in their facilities to maintain their knowledge and skills. The bag-mask ventilation skills check and OSCEs were administered only immediate post-training. Therefore, we were not able to compare HW skills gained relative to skills prior to training. Skills before the course were unlikely to be significant since HBB had not been widely introduced and the structured systematic way of delivering Essential Newborn Care taught in the ECEB module was new to HWs at the time. Laerdal’s mannequin (NeoNatalie) were used for practice and evaluation of trainees rather than assessment of skills in actual clinical practice since the large number of trainees made observation prohibitive [35, 47]. Indeed, several studies have demonstrated the value of simulation-based clinical training in neonatal resuscitation, both in high- and low-resource settings [12, 36]. HW responses may have been hampered because of anxiety as OSCEs were seen as tests/examinations or because they were unfamiliar with skills testing with a mannequin and practice scenarios [36]. We minimized these by encouraging trainees to relax at the beginning of the OSCE and by rehearsing OSCEs and scoring during our preparatory meetings. Furthermore, it is recognized that acquisition of knowledge and skills may or may not be translated into actual clinical behaviour [36, 41]; we do not address this here. Maintenance of HW skills will be discussed in another article, however, how these skills translate into clinical practice and impact newborn morbidity and mortality needs additional study.

## Conclusions

Our simulation-based, hands-on integrated HBB-ECEB-IP training was effective in improving the knowledge and skills of almost 4000 HWs in our target regions. Harmonization and standardization of the course delivery by trainers and having a core team to ensure training fidelity are essential to maintaining high quality while scaling a program. Facility-level clinical practice of various scenarios will be needed to maintain knowledge and skills. Inclusion of HBB, ECEB, and IP into various HW training institutions’ curricula and the introduction of OSCEs into HWs’ annual performance appraisals may help ensure skills retention in essential newborn care. Further, including ECSB into the curriculum will strengthen the course to address expanded issues on neonatal health.

## Supplementary information

**Additional file 1.** MEBCI Training Dataset. Making Every Baby Count Initiative (MEBCI) Training dataset. The dataset used in this work was built from data collected during the training of health works during the Making Every Baby Count Initiative (MEBCI) project implementation in Ghana. The data was collected from November 2014 to July 2017 from trained health workers working in targeted health facilities in the Brong Ahafo, Eastern Volta and Ashanti Regions of Ghana. The dataset is about 1.8 Mb with a total of 3688 observations and 343 variables. The dataset contains demographic data, trainee first attempt performance on the Helping Babies Breathe, and Essential Care for Every Baby Objective Structured Clinical Examinations, and pre and post training results for knowledge tests on Helping Babies Breathe, Essential Care for Every Baby and Infection Prevention.

## Data Availability

The dataset supporting the conclusions of this article is included as an additional file.

## Abbreviations

AR: Ashanti Region
BAR: Brong Ahafo Region
CHN: Community Health Nurses
CHPS: Community-based Health Planning and Services
CIFF: Children’s Investment Fund Foundation
CN: Critical care nurse
DHD: District Health Directorate
ECEB: Essential Care for Every Baby
EmN: Emergency nurses
EnN: Enrolled nurse
ENC: Essential Newborn Care
ER: Eastern Region
FBO: Faith-based organization
GHS: Ghana Health Service
GNNHSAP: Ghana National Newborn Health Strategy and Action Plan
HA: Health assistants
HBB: Helping Babies Breathe
IMCI: Integrated Management of Childhood Illness
IP: Infection Prevention
MA: Medical assistants
MEBCI: Making Every Baby Count Initiative
MHD: Municipal Health Directorate
NMR: Neonatal mortality rate
PA: Physician assistants
PHN: Public health nurses
PN: Paediatric nurses
PON: Peri-operative nurses
RHD: Regional Health Directorate
SD: Standard deviation
TOT: Training of Trainers
VR: Volta Region
WA: Ward assistants
